# Supplementary material for: Phycobiliprotein Peptide Extracts from Arthrospira platensis Ameliorate Nonalcoholic Fatty Liver Disease by Modulating Hepatic Lipid Profile and Strengthening Fat Mobilization
Source: Nutrients. 2023 Oct 27;15(21):4573. doi: 10.3390/nu15214573 (PMC10648092; doi:10.3390/nu15214573)
Supplement: Supplementary file 1 [file nutrients-15-04573-s001.zip › nutrients-2646943-supplementary.pdf]

Supplementary materials

Table S1. Specific peptide sequences of *Arthrospira platensis* phycobiliproteins peptide extracts.

Peptides sequence				
DERVL	LDERVL	LRDLDYY	DPSILDERV	ADQRGKDKCAR
DYYLR	LDYYLR	MFDAFTK	DSQGRFLSS	DPSILDERVLN
EARYL	LRDLDY	MKTPLTE	GIDEINRTF	GDPSILDERVL
GYYLR	MEIILR	NKFPYTT	IRDLDYYLR	GELDRIKSFVT
LDRIK	NKFPYT	RDLDYYL	IVNADAEAR	SPGELDRIKSF
LRDME	RDLDYY	RPDVVSP	LRDLDYYLR	YLSPGELDRIK
LRLIT	SYFDRA	ADAEARYL	LRDMEIILR	AGDPSILDERVL
LRMVT	YIEALK	DMEIILRY	PSILDERVL	LAGDPSILDERVL
LRYVT	YLRMVT	DPSILDER	RTFELSPSW	SPGELDRIKSFVT
NKFPY	DEYLIAG	GIDEINRT	SLLYSDITR	YLSPGELDRIKSF
QGRFL	DLDYYLR	IDEINRTF	SPGELDRIK	NYAADQRGKDKCAR
RDLDY	DMEIILR	NKFPYTTQ	DPSILDERVL	
RTFEL	DSQGRFL	PGELDRIK	DQRGKDKCAR	
RYVTY	EINRTFE	RDLDYYLR	GIDEINRTFE	
SYFDR	GELDRIK	RDMEIILR	GKYLDASAIQ	
ELDRIK	GIDEINR	RPDVVSPG	HGLSGDAAVE	
FDAFTK	GKDKCAR	SILDERVL	LSPGELDRIK	
GELDRI	ILDERVL	SLGVPIGA	SIVNADAEAR	
GELRVR	IRDLDYY	SPGELDRI	SPSWYIEALK	
IRDLDY	LDERVLN	DERVLNGLK	SQADTRGEML	

Table S2. The primer sequences used in this study.

Gene	Sequence (5’ to 3’)	
GAPDH	Forward	GCAAGTTCAACGGCACAG
	Reverse	GCCAGTAGACTCCACGACAT
HSL	Forward	CTGGAGTTAAGTGGGCGCAAG
	Reverse	CAGACACACTCCTGCGCATAGAC
ATGL	Forward	TGACTCGAGTTTCGGATGGAGA
	Reverse	GAAATGCCGCCATCCACATAG
UCP1	Forward	TACCCAGCTGTGCAATGACCA
	Reverse	GCACACAAACATGATGACGTTCC
UCP2	Forward	GCTGGTGACCTATGACCTCATCAA
	Reverse	GTACTGGCCCAAGGCAGAGTTC

Table S3. Significantly different components identified by hepatic lipidomics between PPE_H and HFD groups (VIP value > 2.5, PPE_H vs HFD).

Differential lipids	Formula	RT (min)	m/z (Da)	VIP value	P_value
**CL (24:2/18:0/18:1/22:4)**	C91 H162 O17 P2	7.495	794.5649	2.6517	0.0045
**DLCL (18:1/20:3)**	C47 H85 O15 P2	4.886	951.5369	3.4042	0.0026
**DLCL (18:2/20:4)**	C47 H81 O15 P2	4.047	947.5056	3.1387	0.0307
**GM3(d18:1/24:0)**	C65 H119 O21 N2	8.578	1263.831	2.5132	0.0248
**MLCL (18:2/16:0/18:2)**	C61 H111 O16 P2	8.491	1161.735	5.9458	0.0241
**PI (18:0/18:2)**	C45 H82 O13 N0 P1	6.505	861.5499	6.2786	0.0137
**DG (16:0/16:1)**	C35 H70 O5 N1	7.769	584.5249	2.8386	0.0079
**PE (18:1p/22:4)**	C45 H81 O7 N1 P1	7.748	778.5745	3.4867	0.0157
**TG (16:0/14:0/14:0)**	C47 H94 O6 N1	12.536	768.7076	3.0579	0.0320
**TG (15:0/14:0/16:1)**	C48 H94 O6 N1	12.205	780.7076	3.1611	0.0268
**TG (16:0/14:0/16:1)**	C49 H92 O6 Na1	12.509	799.6786	3.2059	0.01456
**TG (16:0/16:1/18:2)**	C53 H100 O6 N1	12.213	846.7545	2.6953	0.03468
**TG (19:1/18:1/18:1)**	C58 H110 O6 N1	12.631	916.8328	2.6259	0.03612

Table S4. Significantly different components identified by hepatic lipidomics between HFD and Chow groups (VIP value > 2.5, HFD vs Chow).

**Differential lipids**	**Formula**	**RT (min)**	**m/z (Da)**	**VIP value**	**p-value**
**CL (18:2/18:2/18:2/22:6)**	C85 H141 O17 P2	11.008	1495.9650	3.1670	0.0361
**LPG (16:1)**	C22 H42 O9 N0 P1	1.025	481.25720	2.6408	0.0002
**LPMe (18:1)**	C22 H42 O7 N0 P1	1.824	449.26737	3.5592	0.0183
**PE (16:1e/20:5)**	C41 H71 O7 N1 P1	6.471	720.49737	2.6292	0.0001
**CL (18:2/20:3/16:0/20:4)**	C83 H145 O17 P2	11.398	1475.9952	3.2828	0.0047
**PE (16:0/16:1)**	C37 H73 O8 N1 P1	6.501	690.5068	4.0537	0.0005
**TG (22:4/11:1/18:2)**	C60 H106 O6 N2	12.526	950.8045	2.5041	0.0002
**TG (20:1/18:1/22:5)**	C63 H112 O6 N1	13.858	978.84841	2.5874	7.9E-06
**TG (22:4/20:4/22:5)**	C67 H108 O6 N1	12.314	1022.8171	4.4580	3.2E-09

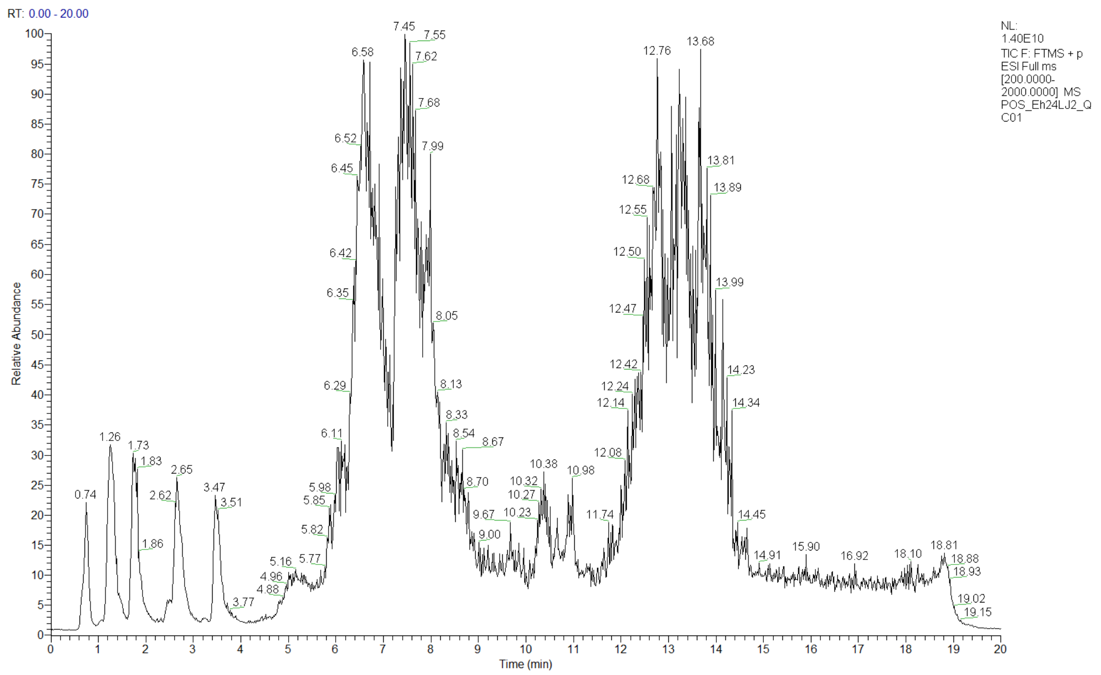

Figure S1. The total ion chromatogram of hepatic lipidomics by LC-MS analysis in positive ion mode.

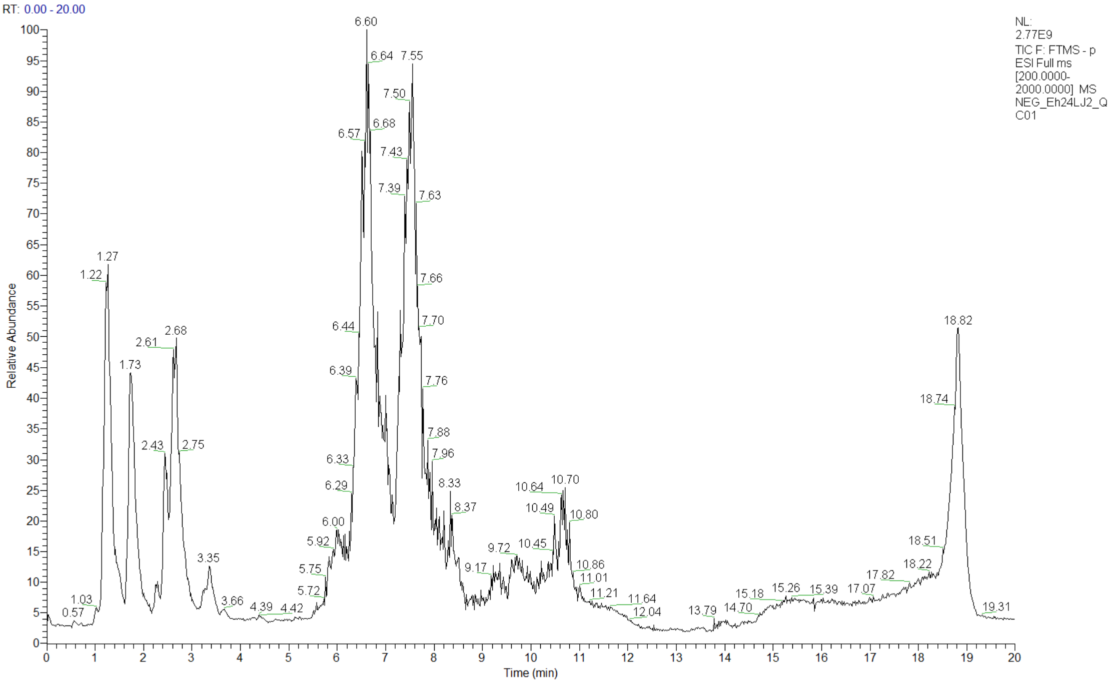

Figure S2. The total ion chromatogram of hepatic lipidomics by LC-MS analysis in negative ion mode.
